# Structural basis of DNA recognition by BEN domain proteins reveals a role for oligomerization in unmethylated DNA selection by BANP

**DOI:** 10.1093/nar/gkae762

**Published:** 2024-09-03

**Authors:** Jiahao Ren, Junmeng Wang, Yanpeng Ren, Yuyang Zhang, Pengshuai Wei, Meng Wang, Yimeng Zhang, Meng Li, Chuyan Yuan, Haipeng Gong, Junyi Jiang, Zhanxin Wang

**Affiliations:** Key Laboratory of Cell Proliferation and Regulation Biology of Ministry of Education, College of Life Sciences, Beijing Normal University, 19 Xinjiekouwai Avenue, Beijing 100875, China; Key Laboratory of Cell Proliferation and Regulation Biology of Ministry of Education, College of Life Sciences, Beijing Normal University, 19 Xinjiekouwai Avenue, Beijing 100875, China; Key Laboratory of Cell Proliferation and Regulation Biology of Ministry of Education, College of Life Sciences, Beijing Normal University, 19 Xinjiekouwai Avenue, Beijing 100875, China; MOE Key Laboratory of Bioinformatics, School of Life Sciences, Tsinghua University, 30 Shuangqing Road, Beijing 100084, China; Beijing Frontier Research Center for Biological Structure, Tsinghua University, 30 Shuangqing Road, Beijing 100084, China; Key Laboratory of Cell Proliferation and Regulation Biology of Ministry of Education, College of Life Sciences, Beijing Normal University, 19 Xinjiekouwai Avenue, Beijing 100875, China; Key Laboratory of Cell Proliferation and Regulation Biology of Ministry of Education, College of Life Sciences, Beijing Normal University, 19 Xinjiekouwai Avenue, Beijing 100875, China; Key Laboratory of Cell Proliferation and Regulation Biology of Ministry of Education, College of Life Sciences, Beijing Normal University, 19 Xinjiekouwai Avenue, Beijing 100875, China; Key Laboratory of Cell Proliferation and Regulation Biology of Ministry of Education, College of Life Sciences, Beijing Normal University, 19 Xinjiekouwai Avenue, Beijing 100875, China; Key Laboratory of Cell Proliferation and Regulation Biology of Ministry of Education, College of Life Sciences, Beijing Normal University, 19 Xinjiekouwai Avenue, Beijing 100875, China; MOE Key Laboratory of Bioinformatics, School of Life Sciences, Tsinghua University, 30 Shuangqing Road, Beijing 100084, China; Beijing Frontier Research Center for Biological Structure, Tsinghua University, 30 Shuangqing Road, Beijing 100084, China; Key Laboratory of Cell Proliferation and Regulation Biology of Ministry of Education, College of Life Sciences, Beijing Normal University, 19 Xinjiekouwai Avenue, Beijing 100875, China; Key Laboratory of Cell Proliferation and Regulation Biology of Ministry of Education, College of Life Sciences, Beijing Normal University, 19 Xinjiekouwai Avenue, Beijing 100875, China

## Abstract

The BEN domain is a newly discovered type of DNA-binding domain that exists in a variety of species. There are nine BEN domain-containing proteins in humans, and most have been shown to have chromatin-related functions. NACC1 preferentially binds to CATG motif-containing sequences and functions primarily as a transcriptional coregulator. BANP and BEND3 preferentially bind DNA bearing unmethylated CpG motifs, and they function as CpG island-binding proteins. To date, the DNA recognition mechanism of quite a few of these proteins remains to be determined. In this study, we solved the crystal structures of the BEN domains of NACC1 and BANP in complex with their cognate DNA substrates. We revealed the details of DNA binding by these BEN domain proteins and unexpectedly revealed that oligomerization is required for BANP to select unmethylated CGCG motif-containing DNA substrates. Our study clarifies the controversies surrounding DNA recognition by BANP and demonstrates a new mechanism by which BANP selects unmethylated CpG motifs and functions as a CpG island-binding protein. This understanding will facilitate further exploration of the physiological functions of the BEN domain proteins in the future.

## Graphical Abstract

**Graphical Abstract:**
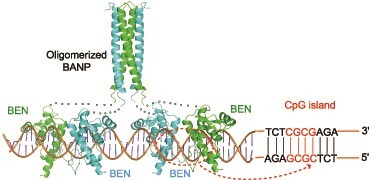

## Introduction

**Figure 1.**
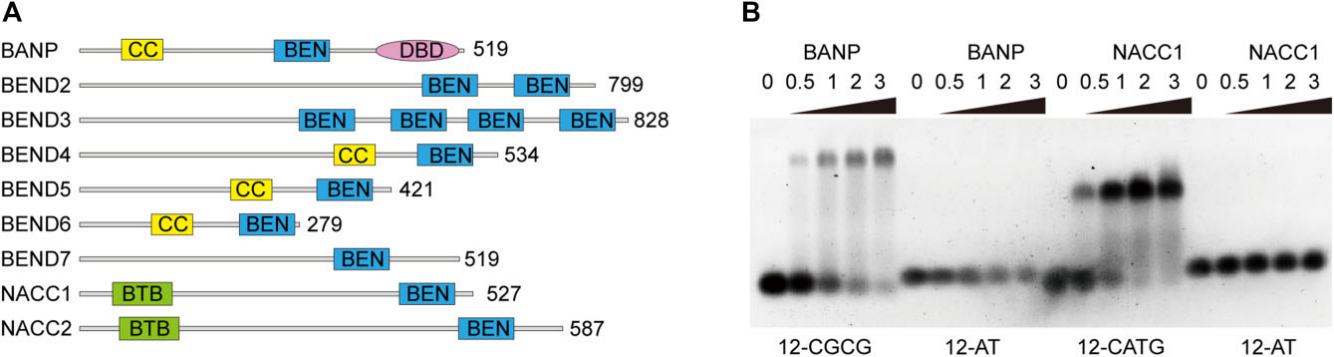
BANP and NACC1 bind DNA with specific motifs. (**A**) Domain architecture of human BEN domain proteins. CC stands for the coiled-coil domain and DBD stands for the DNA-binding domain. (**B**) Electrophoretic mobility shift assay (EMSA) analysis to detect the interaction between the BEN domains from BANP and NACC1 and various double-stranded DNA substrates. The molar ratios of protein to DNA are shown above each channel. 12-CGCG, 12-AT and 12-CATG are the names of the utilized DNA oligos.

The BEN domain, which is named after the exemplar proteins BANP, E5R and NACC1 (also known as NAC1), is found in diverse animal and viral proteins and has been shown to constitute a new class of DNA-binding domain (DBD) (1–4). There are nine BEN domain-containing proteins in humans, including BANP, BEND2–7, NACC1 and NACC2 (Figure 1A). Over the past few years, studies have demonstrated the preferred DNA sequences for some human BEN domain proteins, which can be roughly classified into two groups based on their recognized motifs. The first group of proteins includes NACC1 and NACC2, both of which recognize sequences containing a conserved CATG motif (5). As the CATG motif does not contain any CpG sites, DNA binding by NACC1 and NACC2 is not affected by DNA methylation. NACC1 and NACC2 share 85% sequence similarity and belong to the BTB/POZ protein family, as they both contain a BTB/POZ domain at the N-terminus. NACC1 regulates biological processes such as embryonic development and stem cell pluripotency (6). In neurons, NACC1 interacts with corepressor proteins, including BCL-6, CoREST, HDAC3 and HDAC4, to mediate transcriptional repression (7–9). NACC2 interacts with nucleosome remodelling complexes and represses the transcription of a series of functionally important genes (3).

BANP and BEND3 can be classified into the second group of BEN domain proteins in humans, as both recognize CGCG motif-containing sequences, and their recognition is repelled by methylation of the CGCG motif (10,11). Unmethylated CpG motifs are typically enriched at CpG islands (12); thus, BANP and BEND3 both function as CpG island-binding proteins. BANP contains a coiled-coil domain at the N-terminus, a single BEN domain in the middle and a DBD at the C-terminus. BANP is also known as SMAR1; it was originally identified as a nuclear protein that binds to matrix-associated regions possibly through its C-terminal DBD and functions as a tumour repressor (13–15). Later, by using the single-molecule footprinting method, BANP was identified as a CGCG motif-binding protein, thus linking BANP to CpG islands (10). BANP, which is enriched at a subset of CpG island promoters, is associated with highly expressed genes and functions as an activator of some essential genes. BEND3 contains four BEN domains and associates with heterochromatin; moreover, it represses transcription (16–18). Molecular and biological studies have further demonstrated that BEND3 is a CpG island-binding protein that binds unmethylated CpG motifs through its fourth BEN domain (11). BEND3 facilitates the stable association of PRC2 with bivalent genes to prevent their premature activation during differentiation in embryonic stem cells.

The preferred DNA-binding motifs of the remaining five human BEN domain proteins have not been clearly identified. BEND2 is a key regulator of meiosis during mouse spermatogenesis (19). BEND2 preferentially binds to simple sequence repeats enriched with GA motifs. Moreover, BEND2 has also been shown to bind to a subset of CpG islands in the genome. Therefore, it is not clear whether CpG motifs are also preferred by BEND2. BEND4 and BEND5 work together with the core human pluripotent factors in primordial germ cells and help to mark chromatin boundaries (20). BEND4 and BEND5 binding sites derived from chromatin immunoprecipitation followed by sequencing (ChIP-seq) data contain long consensus sequences. Simplified short motifs measured by other *in vitro* methods may be needed to identify their preferred motifs. BEND6 binds mammalian CBF1 and antagonizes Notch-dependent target activation (21). BEND6 can also bind to CpG motif-containing sequences. However, biochemical studies have shown that BEND6 binding is not sensitive to DNA methylation (22). Therefore, BEND6 may not be a CpG island-binding protein, and its preferred binding motifs remain to be identified. To date, no functional studies on BEND7 have been reported.

The structures of several BEN domain proteins in complex with DNA substrates in *Drosophila* and humans have been solved (4,5,11,22,23), thus demonstrating the sequence preference and DNA recognition mechanisms of these BEN domain proteins. Despite these advances, the DNA recognition mechanisms of several human BEN domain proteins remain to be revealed. The study of human BEN domain proteins is important because binding to CpG islands occurs only for BEN domain proteins in higher vertebrates, such as humans, but not in *Drosophila*, which do not exhibit CpG methylation. In addition, there are some controversies surrounding BANP as a CpG island-binding protein. *In vitro* studies have shown that the BEN domain of BANP is not sensitive to DNA methylation (22), which is inconsistent with the *in vivo* findings that BANP is recruited to CpG islands and repelled by DNA methylation (10). In this study, we solved the crystal structures of the BEN domains of NACC1 and BANP in complex with their bound DNA substrates. Our structures reveal the DNA recognition mechanism of both proteins. Unexpectedly, oligomerization is required for BANP to select the unmethylated CGCG motif over its methylated counterpart, whereas the monomeric BEN domain of BANP alone does not possess this selectivity, thus clarifying the inconsistency observed between *in vitro* and *in vivo* studies. Our study confirms BANP as an actual CpG island-binding protein and reveals a unique mechanism by which BANP senses and selects unmethylated CpG motif-containing DNA substrates.

## Materials and methods

### Protein expression and purification

Constructs containing the BANP or NACC1 segments were prepared by inserting the corresponding segments into a hexa-histidine-SUMO- or hexa-histidine-MBP-tagged pRSFDuet-1 vector. All the proteins were expressed in *Escherichia coli* Rosetta (DE3) cells. The cells were first grown at 37°C until the OD_600_ reached ∼1.2. After cooling at 20°C for ∼0.5 h, 0.1 mM isopropyl β-D-thiogalactopyranoside (IPTG) was added to induce expression overnight. The cells were harvested via centrifugation at 5000 rpm for 10 min.

For full-length BANP and NACC1 proteins, cell pellets were resuspended in initial buffer containing 20 mM Tris (pH 8.0), 500 mM NaCl and 20 mM imidazole, after which they were sonicated for ∼8 min. The soluble fraction of the cells was fractionated via centrifugation of the cell lysate at 18 000 rpm for 50 min. The target proteins were isolated using a nickel-charged HiTrap Chelating FF column from GE Healthcare. The His-MBP tag was cleaved by incubation with the histidine-tagged TEV protease. The His-SUMO tag was cleaved by incubation with the His-tagged ULP1 protease. The protein and protease mixture was diluted and loaded onto a HiTrap Q HP column. The target protein was separated by increasing the NaCl concentration of the low-salt buffer (20 mM Tris, pH 8.0; 100 mM NaCl; 2 mM dithiothreitol (DTT)) from 100 mM to 1 M through a linear gradient.

For the BANP and NACC1 domains, the utilized purification strategies were similar except that in the step of removing the digested tags, a heparin column instead of a Q column was used for the purification. In detail, after protease digestion, the protein and protease mixture was directly loaded onto a heparin column to remove both the bound nucleic acids and tags. The target protein was isolated by increasing the NaCl concentration of the low-salt buffer (20 mM Tris, pH 7.0; 200 mM NaCl; 2 mM DTT) from 200 mM to 2 M through a linear gradient.

After purification on a Q or heparin column, the target protein was further purified on a HiLoad 200 16/600 gel filtration column with buffer containing 20 mM Tris (pH 7.0), 200 mM NaCl and 2 mM DTT. The eluted target protein was concentrated and stored in a −80°C freezer.

All the DNA oligos used were chemically synthesized. The sequences of one chain of these sequences are as follows: 12-CGCG, ATCTCGCGAGAT; 12-AT, TTTTATATATAA; 12-CATG, ATTACATGTAAT; 12-mCGCG, ATCT(mC)GCGAGAT; 12-CGmCG, ATCTCG(mC)GAGAT; and 12-mCGmCG, ATCT(mC)G(mC)GAGAT.

### ITC measurements

Isothermal titration calorimetry (ITC)-based experiments were conducted at 20°C using a MicroCal iTC200 instrument. The purified wild-type or mutant proteins and the DNA substrates were dialysed overnight at 4°C in buffer containing 20 mM Tris (pH 7.5), 200 mM NaCl and 2 mM β-mercaptoethanol. During the experiments, protein and DNA samples were diluted with the same dialysis buffer to the proper concentrations before use. Binding analysis was performed by titrating the DNA ligands into the protein samples. The calorimetric titration curves were analysed with Origin software using the one binding site model algorithm. Each ITC measurement was performed at least twice.

### EMSA analysis

Double-stranded DNA (50 pmol) was mixed with increasing amounts of recombinant BEN domains from BANP or NACC1 in buffer containing 20 mM Tris (pH 7.0), 200 mM NaCl and 2 mM DTT and incubated at 4°C for 10 min. The mixture was subsequently loaded onto a 1.2% agarose gel in TAE buffer (40 mM Tris-acetate, 1 mM EDTA, pH 8.0) for electrophoresis and detected via ethidium bromide staining. All of the EMSA experiments were repeated at least three times.

### Crystallization and structure determination

Crystallization of the BEN domain of BANP in complex with the target DNA was performed via using the hanging-drop, vapour-diffusion method by mixing equal volumes of protein and the well solution. The 12-bp palindromic DNA sequence with a cytosine overhang at the 5′ end was used for crystallization. One strand of the DNA sequence was 5′-CATCTCGCGAGAT-3′. The protein/DNA complex was prepared by mixing the BEN domain of BANP (residues 205–325) with the target DNA at a molar ratio of 2:1.1. Crystals of the complex were grown with a solution containing 0.02 M citric acid, 0.08 M bis-tris propane (pH 8.8) and 16% (w/v) polyethylene glycol 3350 at 4°C. Crystals were flash frozen with crystallization buffer containing 10% 2,3-butanediol as the cryoprotectant.

For the crystals of the DNA-bound BEN domain of NACC1, 10-bp palindromic DNA bearing a thymine overhang at the 5′ end with the sequence 5′-TTTACATGTAA -3′ was used. The BEN domain of NACC1 (residues 341–477) and the target DNA mixed at a molar ratio of 2:1.1 were used for crystallization. Crystals were obtained with buffer containing 0.1 M imidazole at a pH of 7.0 and 12% (w/v) polyethylene glycol 20 000. The crystals were frozen in crystallization buffer with the addition of 25% glycerol as the cryoprotectant.

All of the datasets were collected at the Shanghai Synchrotron Radiation Facility beamlines in China at a temperature of −196°C. Datasets for the crystals of the selenomethionine-labelled BEN domain of NACC1 were collected at the BL18U1 beamline at a wavelength of 0.97853 Å. Datasets for the crystals of the DNA-bound BEN domain of BANP were collected at the BL18U1 beamline at a wavelength of 0.97915 Å. The datasets were processed via the program HKL2000 (24). The structure of the BANP/DNA complex was solved via the molecular replacement method by PHENIX (25) using the AlphaFold2 (26)-generated BANP model as the input model. The structure of the NACC1/DNA complex was solved via the single-wavelength anomalous diffraction method by using the anomalous signals from the selenomethionine-labelled crystals. The initial models of both complexes were manually rebuilt through COOT (27) and further refined via PHENIX.

### Oligomeric state analysis by the glutaraldehyde-mediated cross-linking method

Purified full-length BANP was dialysed overnight at 4°C in buffer containing 20 mM N-2-hydroxyethylpiperazine-N-2-ethane sulfonic acid (HEPES), pH 7.5, 200 mM NaCl and 2 mM DTT. Then, the cross-linking reagent glutaraldehyde was added to the protein sample to a final concentration of 0.06%. After incubation on ice for 10 min, the cross-linking reaction was quenched by adding 1 M Tris (pH 7.5) buffer to the sample to a concentration of 100 mM. After centrifugation at 14 000 rpm at 4°C for 10 min, the cross-linked sample was loaded onto a Superdex 200 10/300 GL column (GE Healthcare) equilibrated with buffer containing 200 mM NaCl, 20 mM Tris (pH 7.5) and 2 mM DTT. Eluted fractions were collected and further analysed by sodium dodecyl sulfate–polyacrylamide gel electrophoresis.

### Molecular dynamics simulation

The molecular dynamics analysis was based on the crystal structure of BANP solved in this study (8YZT). For the cytosine-methylated model, the methylated cytosines were first accessed from AMBER-Hub (http://amberhub.chpc.utah.edu) and then superimposed on each of the original bases for substitution. Tleap (28) was used to add missing atoms to the model. For water box preparation, the model was first rotated to align the principal axes to the box axes, and then water molecules and Na^+^ ions were added to form a 98 × 98 × 98 nm^3^ isometric square box. The Amber-ff14SB parameter set (29) was used as the force field for all protein molecules, and Amber-OL15 (30) was used for all DNA bases. For the noncanonical methylated cytosines, the modified force field parameter set (31) was used as a supplement. The TIP3P water model was used for water box preparation. For simulation, we use Amber 18 (32) to perform molecular dynamics simulations throughout the experiments. Each model was first minimized for a maximum of 10 000 steps via steepest descent and conjugate gradient algorithms. The system was gradually heated to 310 K with 1 ns of restrained simulation, followed by a 4-ns simulation under the NVT ensemble and a 6-ns simulation under the NPT ensemble for pre-equilibration. The production run was performed under the NVT ensemble for 200 ns with a time step of 2 fs. For all the simulations, we used a Langevin thermostat with a collision frequency of 3 ps^−1^. We used van der Waals and short-range electrostatic interaction cut-offs of 1 nm. For each group of interest, we performed 10 independent replicates.

The trajectories were saved as snapshots every 40 ps of simulation. For the root-mean-square deviation (RMSD) and root-mean-square fluctuation (RMSF) analyses, we used MDAnalysis (33). Each snapshot was first aligned to the initial model based on the protein backbone, and the RMSD of each snapshot and the RMSF of each position of residues along one trajectory were subsequently computed. For hydrogen bonding network analysis, we used MDTraj (34) to identify all Baker–Hubbard hydrogen bonds for each snapshot. The hydrogen bonds involved in DNA–protein interactions were recorded to analyse the stability of the interactions. The total number of formed hydrogen bonds and the formation frequency of each DNA–protein interaction pair were computed for differential analysis.

## Results

### BANP and NACC1 can both recognize their preferred DNA sequences

As BANP and NACC1 each contain a single BEN domain (Figure 1A), we hypothesized that their BEN domains are responsible for DNA recognition. We expressed the full-length and single domains of both proteins. Through EMSA, we verified that BANP can bind to a CGCG-containing DNA substrate, whereas NACC1 prefers a CATG-containing DNA sequence (Figure 1B). BANP and NACC1 did not bind to randomly selected AT-rich DNA (Figure 1B). We also tested the DNA-binding ability of the DBD of BANP and found that it does not bind the CGCG-containing DNA used in this study (Supplementary Figure S1A).

### Crystal structure of the NACC1/DNA binary complex

**Figure 2.**
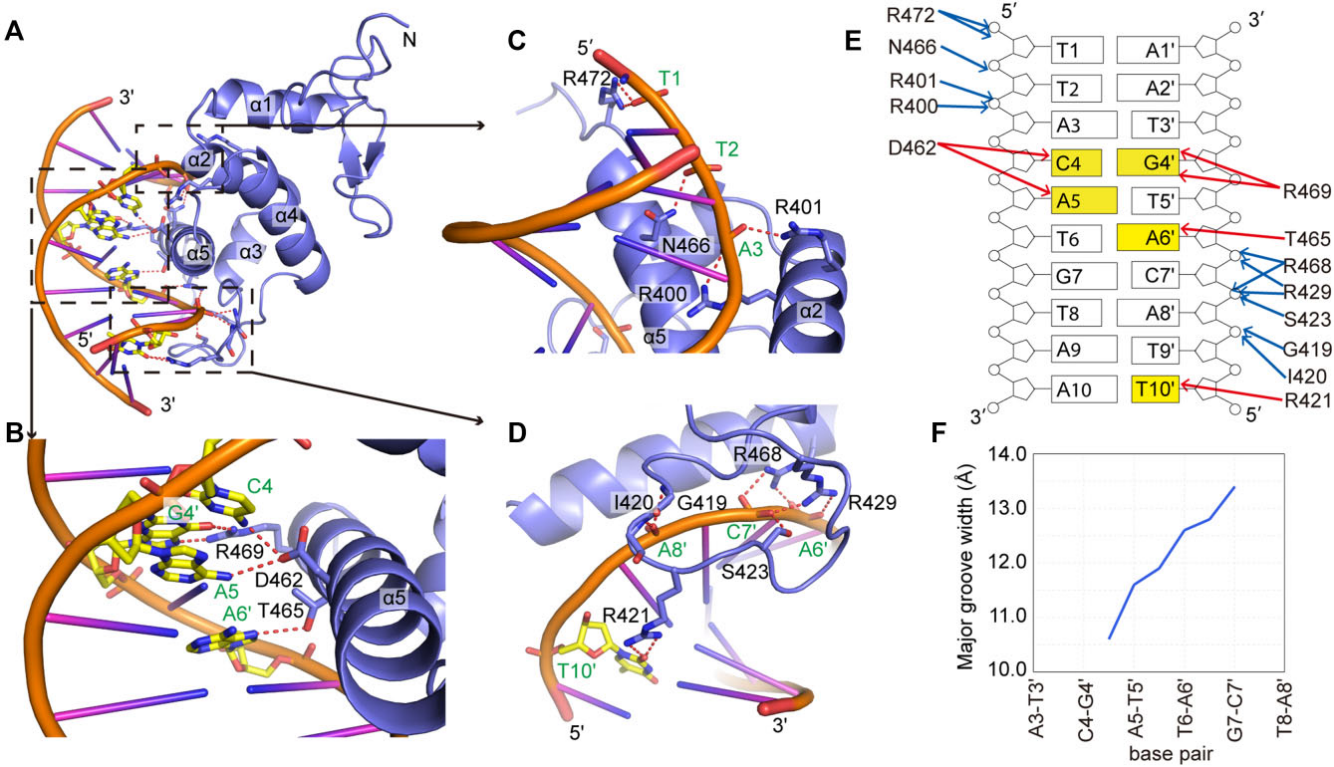
Overall structure of the NACC1–BEN/DNA complex. (**A**) An overall view of the complex of the BEN domain of NACC1 with its bound DNA substrate. (**B**–**D**) Details of the interaction between the BEN domain of NACC1 and the DNA substrate. The DNA bases recognized by NACC1 are shown in the stick model. Hydrogen bonds are shown as dotted lines. (**E**) A schematic representation of the hydrogen bonds formed between NACC1 and the DNA substrate. The recognized DNA bases are highlighted. (**F**) Curve plot of the major groove width of the substrate DNA as measured by CURVES+ (35).

To clarify the molecular basis of DNA recognition by NACC1, we solved the crystal structure of the BEN domain of NACC1 in complex with bound 10-bp CATG-containing DNA with a thymine overhang at the 5′ end at a resolution of 2.3 Å (Supplementary Table S1). In the structure, the BEN domain of NACC1 contains two β-strands at the N-terminus and five α-helices that fold into a globular structure (Figure 2A). In this complex, one NACC1 molecule binds one double-stranded DNA molecule. Sequence-specific DNA recognition is mediated primarily by the C-terminal helix α5 of the NACC1 BEN domain, which inserts into the major groove and contacts the bases inside (Figure 2A). Asp462 from α5 forms a hydrogen bond each with the bases of C4 and A5 from one chain of DNA. Thr465 and Arg469 from α5 recognize bases from the other chain of DNA, with Thr465 forming a hydrogen bond with A6′ and Arg469 forming a pair of hydrogen bonds with G4′, respectively (Figure 2B). The phosphate backbones on both sides of the major groove in which α5 inserts are also contacted by the BEN domain. The long side chains of Arg400 and Arg401 from α2 and Asn466 and Arg472 from α5 interact with the phosphate backbone of A3, T2 and T1 from one side of the phosphate backbone (Figure 2C). The side chains of Arg468 from α5 and Arg429 and Ser423 from the loop between α3 and α4 contact the phosphate backbone of A6′ and C7′ from the other side (Figure 2C). In addition, the G419–Ile420 segment from the loop between α3 and α4 forms a pair of hydrogen bonds with the phosphate backbone of A8′ through the main chain atoms (Figure 2D). Notably, nearly half of the loop between α3 and α4 hangs over the neighbouring minor groove, with the long side chain of Arg421 from this loop inserting into the narrow minor groove and forming a pair of hydrogen bonds with the base of T10′ (Figure 2D). Overall, NACC1 covers a length of 10 bp on the DNA substrate. Within the DNA substrate, bases from one or both strands of the CAT motif are specifically recognized (Figure 2E), which is consistent with the *in vitro* findings (5). The insertion of the α5 helix widens the CATG-containing major groove by 1–2 Å (Figure 2F).

### Mutational analysis of the DNA-binding residues of NACC1

**Figure 3.**
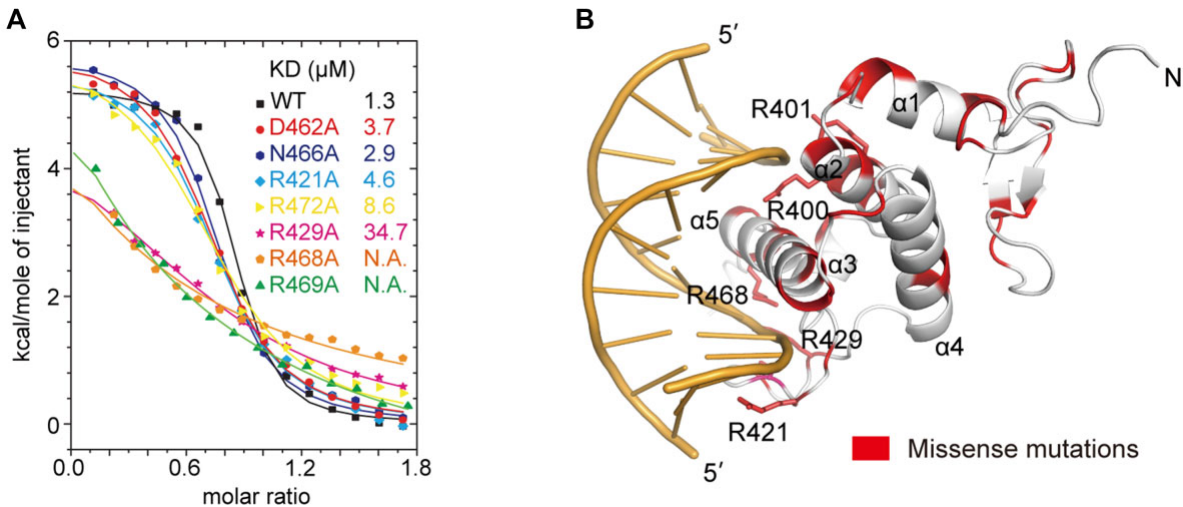
Mutation analyses of the BEN domain of NACC1. (**A**) ITC-based binding analyses of the wild-type (WT) and mutated NACC1 BEN domains with the DNA substrate. Dissociation constants (*K*_D_) are shown as insets. N.A., not available. (**B**) Positions of the missense mutations recorded in the COSMIC database were mapped to the structure of the BEN domain of NACC1. The mutation sites are highlighted. The DNA-binding residues are shown in the stick model.

We subsequently generated a series of mutations that disrupted the interactions between the NACC1 BEN domain and its target DNA. The wild-type NACC1 BEN domain binds CATG-containing DNA with a molar ratio of ∼1:1 and a dissociation constant (*K*_D_) of 1.3 μM. Mutations in the BEN domain that disrupt base-specific recognition, such as D462A and R421A, reduced the binding affinities to 28–36% of that of the wild-type protein, as measured by ITC-based analyses (Figure 3A and Supplementary Table S2). The R469A mutation almost completely abolished the interaction between NACC1 and the target DNA, further verifying the role of Arg469 in base-specific recognition (Figure 3A). Mutations in the residues that contact the phosphate backbone also reduce the binding of the NACC1 BEN domain to the target DNA. Notably, the R429A and R468A mutations exhibited dramatically impaired binding, thus decreasing the binding affinity to <5% of that of the wild-type protein (Figure 3A). Previous studies have revealed the oncogenic roles of NACC1, including the regulation of cancer cell cytokinesis and tumour suppressor inactivation (36,37). Deposited data in the Catalogue Of Somatic Mutations In Cancer (COSMIC) database (38) revealed >50 types of mutations within the BEN domain of NACC1. Importantly, many of these mutations occurred in the DNA-binding residues shown in our structure (Figure 3B). Mutations such as R400G, R400W, R401Q, R421H, R429W, R468C and R468H are expected to reduce the interaction between NACC1 and the target DNA, thus resulting in a weakened chromatin association for NACC1 that is associated with carcinogenesis.

### Crystal structure of the BANP/DNA binary complex

**Figure 4.**
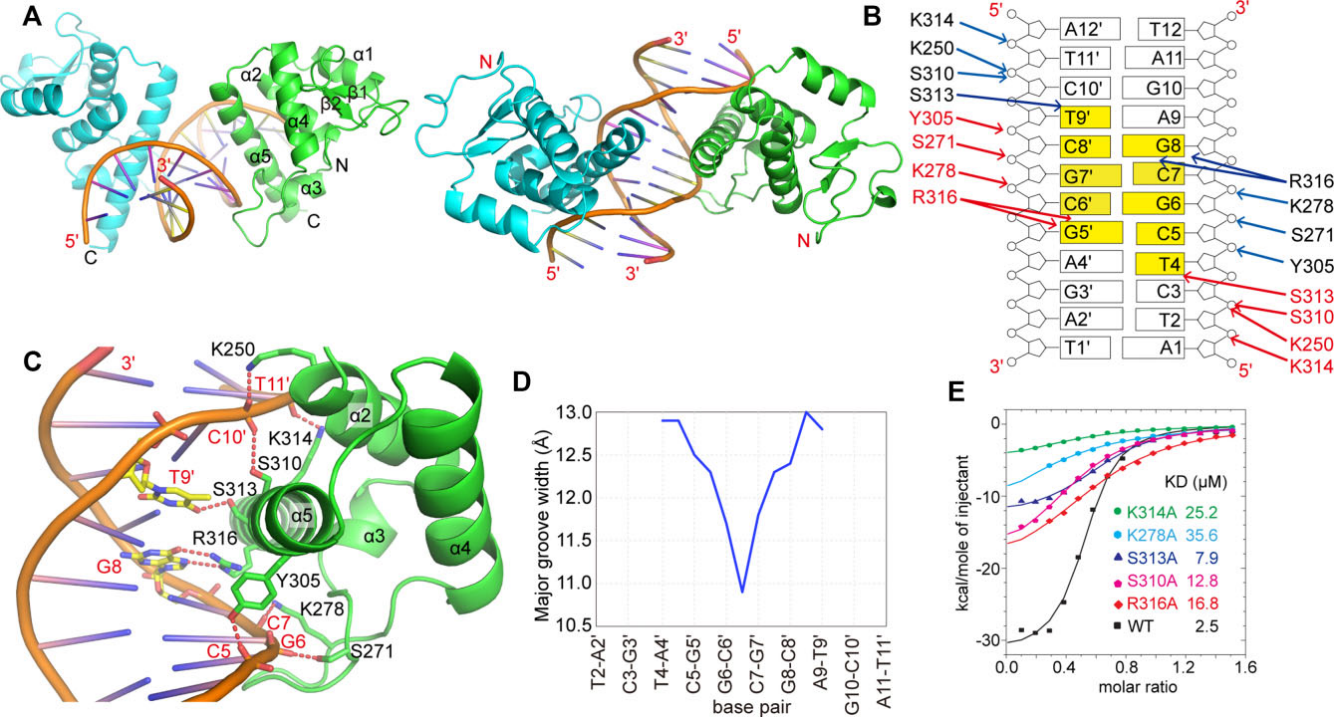
Structural basis of DNA recognition by the BEN domain of BANP. (**A**) Two views of the BANP–BEN/DNA complex. (**B**) A schematic representation of the interactions between BANP and the target DNA. The CGCG motif and the specifically recognized T9′ and T4 regions are highlighted. Interactions with DNA from two BANP molecules are shown. (**C**) Structural details of the contacts between BANP and the target DNA. Hydrogen bonds are shown as dotted lines. The recognized DNA bases are shown in the stick model. (**D**) A plot showing the major groove widths of the DNA in the structure as measured by CURVES+ (35). (**E**) Dissociation constants (*K*_D_) between wild-type (WT) or various mutants of BANP and a CGCG motif-containing DNA substrate measured via ITC. The dissociation constants are shown as insets.

BANP has been shown to preferentially bind CGCG motif-containing sequences (10), which was confirmed by our preliminary analysis (Figure 1B). To understand the molecular basis of DNA recognition by BANP, we solved the crystal structure of the BEN domain of BANP in complex with 12-bp palindromic CGCG-containing DNA with a cytosine overhang at the 5′ end at a resolution of 2.6 Å (Supplementary Table S1). In the complex structure, the BEN domain of BANP folds similarly to the BEN domain of NACC1, with two N-terminal β strands followed by five α-helices (Figure 4A). Two BANP molecules bind one double-stranded DNA in a head-to-head manner, with each recognizing the target DNA from the opposite direction (Figure 4A). As both BANP molecules interact with the target DNA in a similar manner, we only show the DNA binding for one BANP molecule below. In detail, the long C-terminal helix α5 of the BEN domain is mainly responsible for DNA recognition, as it inserts deeply into the CGCG-containing major groove and makes close contacts with its neighbouring bases and the phosphate–sugar backbones of the target DNA. Sequence-specific interactions for the target DNA could only be observed for G8 from one strand and T9′ from the complementary strand, in which the bases form hydrogen bonds with Arg316 and Ser313 from α5 of the BEN domain, respectively (Figure 4B and C). In addition to base-specific recognition, the phosphate backbones on both sides of the major groove are also in contact with the BEN domain. Lys314 and Ser310 from α5 and Lys250 from α2 of the BEN domain each form a hydrogen bond with the phosphate backbone of T11′ and C10′ from one side of the DNA strand (Figure 4C). Tyr305 from α5 and Ser271 and Lys278 from the loop between α3 and α4 interact with the phosphate backbone of C5, G6 and C7, respectively (Figure 4C). Overall, the BANP BEN domain contacts the target DNA over a 6-bp footprint, with the C-terminal α5 inserted inside the major groove and with α2 and the loop between α3 and α4 holding both rims of the major groove (Figure 4B). BANP binding slightly distorts the target DNA by narrowing the major groove it inserts into (Figure 4D), which is different from NACC1, which slightly widens the major groove it binds (Figure 2F). Nevertheless, such distortion of the major groove does not induce noticeable bending of the target DNA, as measured by CURVES+ (35). Notably, in this complex structure, the loop between α3 and α4 of the BEN domain also extends to cover part of the neighbouring minor groove of the target DNA, but no base from the minor groove is specifically recognized.

We next generated a series of point mutations to disrupt the interaction between BANP and the target DNA. ITC-based measurements demonstrated that the wild-type BEN domain of BANP interacted with the target DNA at a molar ratio of ∼2:1 and with a dissociation constant of 2.5 μM (Figure 4E and Supplementary Table S2). Mutations of the BANP BEN domain that either disrupt base recognition or disrupt phosphate backbone recognition reduced the binding affinities between BANP and the target DNA (Figure 4E). This result indicates that DNA recognition by the BANP BEN domain is the combined result of all of these base- and backbone-mediated interactions.

The aberrant expression of BANP has been closely associated with breast cancer and acute myeloid leukaemia; thus, BANP has the potential to be a prognostic/diagnostic marker and therapeutic target (39,40). The COSMIC database records >70 types of mutations within the BEN domain of BANP (38) (Supplementary Figure S1B). Although the impact of most of these mutations is unknown, missense mutations in DNA-binding residues, such as S271L, K314N and R316C (Supplementary Figure S1B), are expected to disrupt the DNA binding of BANP, which may be related to its carcinogenesis.

### Full-length BANP is sensitive to DNA methylation but the BEN domain alone is not

**Figure 5.**
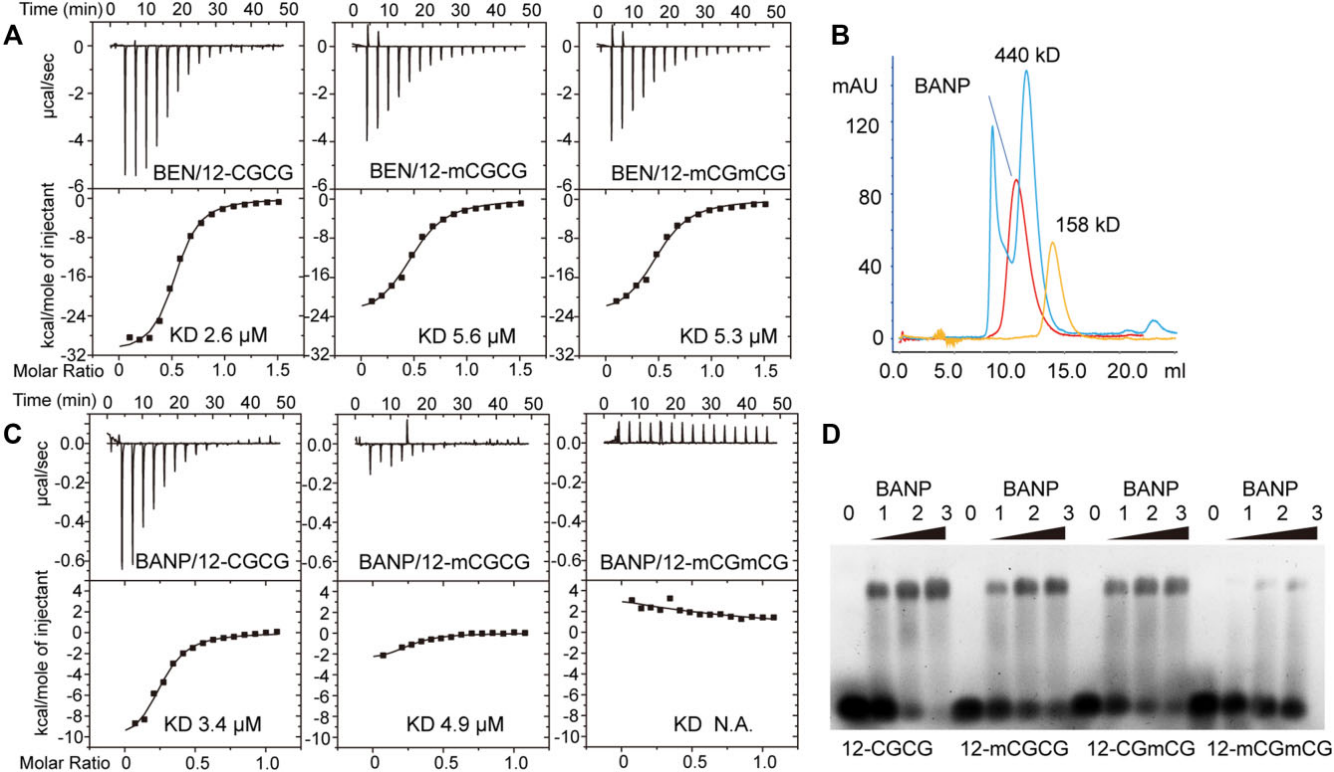
Full-length BANP is sensitive to DNA methylation. (**A**) ITC titration curves of the BEN domain of BANP with various DNA ligands. 12-CGCG, 12-mCGCG and 12-mCGmCG are short for 12-CGCG DNA without methylation, with methylation on one CpG motif and with methylation on both CpG motifs, respectively. Dissociation constants (*K*_D_) are shown as insets. (**B**) Gel filtration profiles of BANP, ferritin (440 kDa) and aldolase (158 kDa). (**C**) ITC-based measurements of full-length BANP with various DNA substrates. The utilized DNA substrates are the same as those in panel (A). The dissociation constants are shown as insets. N.A., not available. (**D**) EMSA analysis of full-length BANP with various DNA substrates.

It has been shown that BANP prefers CGCG motif-containing DNA sequences, and the binding affinities of BANP decrease dramatically when the cytosines in the CGCG motifs are methylated (10). Thus, BANP is considered a CpG island-binding protein that occupies a subset of the CGCG motif-enriched CpG islands in the genome (10). However, structural studies could not support the tandem CG motif preference of BANP, as only one CpG was recognized in the structure of the BANP BEN domain complexed with CGCG-containing DNA (22). In addition, *in vitro* biochemical studies have shown that the BANP BEN domain alone is a methylation-insensitive DBD (22), which is inconsistent with the *in vivo* findings. To address these controversies, we first measured the binding affinities of the BANP BEN domain for CGCG-containing DNA substrates with or without methylation via ITC. We found that the BANP BEN domain showed slightly reduced binding affinities towards hemi- or fully methylated CGCG-containing DNA substrates compared with that obtained by using the unmethylated counterpart (Figure 5A and Supplementary Table S2), which is consistent with previous *in vitro* studies (22). Our structure also revealed that a single BEN domain of BANP can recognize only one CpG motif (Figure 4B). However, in our structure, two BEN domains recognize both CpG sites in the CGCG motif simultaneously (Figure 4A), indicating that, to recognize the CGCG motif, oligomeric BEN domains may be needed. We subsequently determined that full-length BANP contains a coiled-coil domain at the N-terminus, which is a well-known structural element that can mediate homo- or hetero-oligomerization of the target protein (41). Oligomerized full-length proteins may behave differently from the monomeric BEN domain that is used in *in vitro* studies. Afterwards, we purified the full-length BANP protein. Using gel filtration analysis, we found that full-length BANP exists as an oligomer (Figure 5B). Through a cross-linking method, we verified that full-length BANP exists mainly as a tetramer in solution (Supplementary Figure S1C and D). We then repeated the DNA binding analyses via ITC. We found that full-length BANP binds the unmethylated CGCG-containing DNA substrate with a molar ratio of ∼4:1 and a dissociation constant similar to that obtained using the BEN domain alone (Supplementary Table S2). For the hemi-methylated DNA substrates, full-length BANP showed slightly decreased binding affinity. Concomitant with the decreased binding affinity, the reaction heat generated during the titration of the hemi-methylated DNA substrate decreased to ∼1/4 of that generated when the unmethylated DNA counterpart was used as the titrant (Figure 5C and Supplementary Table S2). Unexpectedly, the binding affinity between full-length BANP and the fully methylated DNA substrate could not be detected, as the generated reaction heat decreased to the level of the titration background (Figure 5C). To further verify these results, we repeated the binding analyses via EMSA. Similarly, we found that full-length BANPs exhibited slightly reduced binding affinities for hemi-methylated CGCG-containing DNA substrates and almost no binding affinities for fully methylated DNA substrates (Figure 5D). These results are dramatically different from those generated using only the BEN domain of BANP as the substrate; however, they are perfectly consistent with the *in vivo* findings, wherein full-length BANP was used for all of the analyses (10). Therefore, for BANP, oligomerization may play an important role in selecting the sequence of the DNA substrates.

### Oligomerization is required for BANP to select unmethylated CGCG motifs

**Figure 6.**
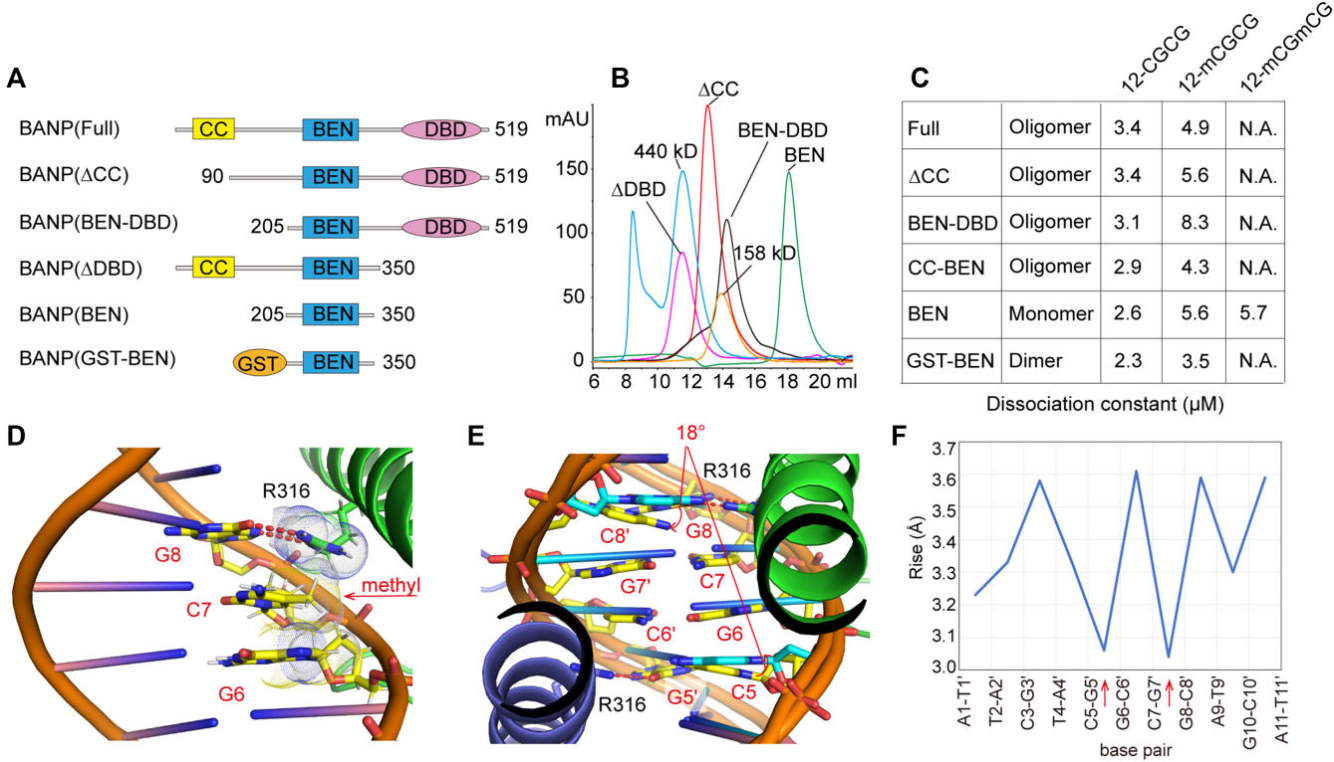
Oligomerization is required for BANP to sense DNA methylation. (**A**) Constructs of BANP used for binding analysis. (**B**) Gel filtration profiles of BANP without the coiled-coil domain (ΔCC), with only the BEN and DNA-binding domains (BEN-DBD), without the DNA-binding domain (ΔDBD) and with only the BEN domain (BEN) are shown, with the profiles of ferritin (440 kDa) and aldolase (158 kDa) as references. (**C**) ITC-based measurements of dissociation constants between various lengths of BANPs and DNA substrates. 12-CGCG, 12-mCGCG and 12-mCGmCG are short for 12-CGCG DNA without methylation, with methylation on one CpG motif and with methylation on both CpG motifs, respectively. N.A., not available. (**D**) Structural model showing that the added methyl group on the C7 base clashes with the G6 base and the guanidino group of Arg316. The arrow indicates the position of the methyl group. (**E**) Structural details of two BEN domains recognizing one CGCG motif, with a superimposed ideal B-form DNA as a reference. C8′ and C5 deviate from the ideal G–C plane by an angle of 18°. (**F**) Plot of spacings (labelled as rise) between neighbouring base pairs of the DNA substrate bound by BANP.

To verify regions that are important for full-length BANPs to sense DNA methylation, we expressed a series of BANP truncations and then checked their oligomeric states by the gel filtration method first (Figure 6A). We found that BANP with the N-terminal coiled-coil domain but without the C-terminal DBD still exhibited an oligomer (Figure 6B), revealing the role of the coiled-coil domain in mediating oligomerization. Unexpectedly, BANP without the coiled-coil domain but with the DBD was also an oligomer (Figure 6B). Further truncation revealed that BANP with only the BEN and DNA-binding domains still appeared as an oligomer (Figure 6B). These findings verified that the C-terminal DBD also plays a role in mediating the oligomerization of BANP. As a control, the BANP BEN domain alone served as a monomer (Figure 6B). To further test the role of BANP oligomerization, a glutathione S-transferase (GST) tag was fused at the N-terminus of the BEN domain of BANP to generate a GST–BEN fusion protein. As GST forms a dimer in solution, GST–BEN also exists as a dimer (Supplementary Figure S1E). We then measured the binding affinities of these BANP truncations with CGCG-containing DNA substrates with/without methylation via ITC. We found that only the monomeric BEN domain of BANP is not sensitive to DNA methylation. BANPs with the coiled-coil domain, the DBD or even the artificially fused GST tag all retained their sensitivity to DNA methylation; they did not bind fully methylated CGCG-containing DNA substrates at all (Figure 6C and Supplementary Table S2). Thus, for BANP, oligomerization is not only necessary but also required to select unmethylated DNA substrates.

### Structural basis of unmethylated CGCG selection by BANP

To identify the molecular basis underlying the selection of the unmethylated CGCG motif by the BANP BEN domain, we modelled a methyl group at the fifth carbon position of the base of C7 in one DNA strand. With the hydrogen atoms shown in the model, we found that the hydrogen atoms from the added methyl group of the DNA molecule clashed with both the guanidino group of Arg316 from the BEN domain and the adjacent guanine ring of the base of G6 (Figure 6D), thus providing a molecular explanation for the preference towards unmethylated CpG motif-containing DNA. However, why the monomeric form of the BANP BEN domain can tolerate DNA methylation is unknown. One explanation for this scenario could be that, to accommodate the added methyl group, either the long side chain of the arginine or the bases, including the methylated C7 and its adjacent G6, need to shift some distance to avoid clashing with one another. For oligomeric BANP, two or more BEN domains recognize one CGCG motif simultaneously in a coordinated manner. As shown in our solved structure (Figure 6E and Supplementary Figure S3), Arg316 recognition of the base of G8 forces the complementary base of C8′ to deviate from the ideal G–C plane. The base of C8′ bends with a buckle angle of −10° and rotates with a propeller angle of −18°, as calculated by w3DNA (42), resulting in a conformation that is closer to the previous base pair. A similar scenario is also observed for the base of C5, which is symmetrically related to C8′ (Figure 6E). When the CGCG motif is recognized by two BEN domains simultaneously, the combined effects of recognition by two arginines from opposite directions of the double-stranded DNA result in compression of the CGCG motif (Figure 6E). Consistent with this speculation, the spacing between the C8′–G8 base pair and its previous G7′–C7 base pair is 3.04 Å, which is much shorter than the average spacing of 3.34 Å between two standard B-form base pairs (Figure 6F). A similar result is also observed for the symmetrically related C5–G5′ base pair. The methylated CGCG motif resists compression, thus destabilizing the recognition of this motif by two BANPs.

### Molecular mechanism of unmethylated DNA preference by BANP revealed by molecular dynamics simulation

**Figure 7.**
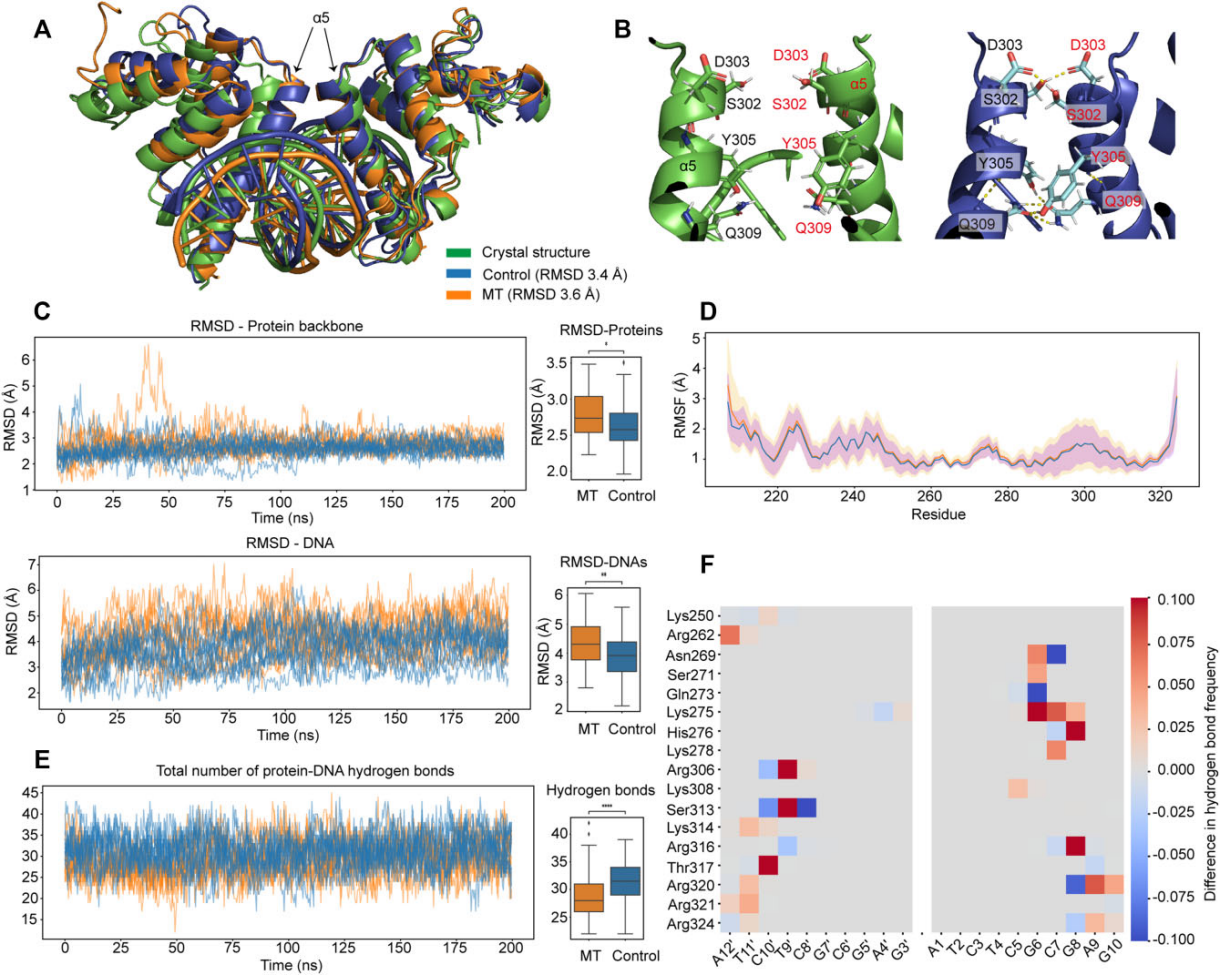
Molecular dynamics simulations of the interaction between BANP and the DNA substrates. (**A**) Alignment of the structures of the control group and the MT group with the crystal structure of the BANP/DNA complex. (**B**) Residues at the N-termini of α5 from two BANP molecules do not have direct interactions in the crystal structure (left panel) but form a hydrogen bonding network in the MT groups (right panel). (**C**) Left: RMSD curves of the structures of the control group and MT group from 20 simulation trajectories compared with the crystal structure. The RMSDs of the protein backbones and the DNAs are listed in the top and bottom panels, respectively. Right: Boxplots of the RMSD differences. For each replicate, we collected 5 evenly spaced snapshots between 100 and 200 ns into the simulation, resulting in 50 snapshots per group for comparisons. Statistical analysis was performed using the Mann–Whitney–Wilcoxon test. (**D**) RMSF curves of each protein residue of the structures of the control and MT groups compared with the crystal structure. (**E**) Left: Curves of the numbers of the protein–DNA hydrogen bonds counted at each snapshot. Right: Boxplot of the difference in hydrogen bond count. Statistical analysis was performed using the Mann–Whitney–Wilcoxon test. (**F**) The difference in hydrogen bond formation frequency between the MT group and the control group.

To further understand the dynamic process of DNA recognition by oligomeric BANP, we performed molecular dynamics simulations on the interactions between two BEN domains of BANP and one CGCG-containing DNA substrate with or without cytosine methylation, using the crystal structure of the BANP/DNA complex or that with artificially modelled methylate groups as the initial structures. BANPs with unmethylated and fully methylated CGCG-containing DNA substrates were designated as the control and the methylated (MT) groups, respectively. During simulations, we detected a notable conformational change that robustly occurred in both groups in all of the 20 replicate runs. Specifically, the closely positioned DNA-binding α5 helices from both BEN domains slide towards each other, reaching RMSDs of 3–4 Å from the initial states (Figure 7A). By measuring the distance between the Ser302 residues and the angle between α5 helices of the two BEN domains (Supplementary Figure S2A), we found that these conformational changes were mainly mediated by the hydrogen bonds formed between the N-terminal parts of the α5 helices. In particular, residues Ser302, Asp303, Tyr305 and Gln309 from α5 helices of both BANP molecules form a dense hydrogen bonding network (Figure 7B), which, although absent in the crystal structure, thermodynamically favours the simultaneous binding of two BANP molecules to one CGCG motif.

Although the two groups converged at their respective final conformations as described above, the MT group experienced a significantly larger conformational change than the control group, as measured by the RMSDs of the backbone atoms of the proteins and DNAs from a series of sampled snapshots (Figure 7C), in which the DNA substrate was likely to be further distorted from its natural form to accommodate the additional methyl groups. In addition, the per-residue structural fluctuation indicates a slightly higher variance of the α5 helix for the MT group, despite the subtle difference in general (Figure 7D). In order to further understand the influence of cytosine methylation on the interaction between BANP and the target DNA, for both the control and MT groups, we counted the hydrogen bonds between BANP and the DNA substrate in the total of 200 snapshots evenly sampled from the trajectories with an interval of 20 ns. Those results suggest that the control group forms a significantly larger number of hydrogen bonds, indicating a higher stability for its protein–DNA interaction (Figure 7E). Moreover, we evaluated the pairwise hydrogen bond formation in each snapshot and computed the interaction frequency for every candidate residue pair. Specifically, we observed a highly stable hydrogen bonding pattern in the unmethylated control group, exemplified by Arg306–T9′, Ser313–T9′, Lys314–T11′, Thr317–C10′ and Arg321–A12′ in one chain as well as Arg316–G8 and Arg320–A9 in the other chain (Supplementary Figure S2B), many of which are present in >50% of the simulation snapshots. The hydrogen bonding network in the MT group, however, is rather unstable (Supplementary Figure S2B), in that many of the aforementioned interactions are frequently disrupted and the hydrogen bonds are often either broken or shifted towards the neighbouring residues or base pairs. Similarly, the heatmap of the difference between the control and MT groups (Figure 7F) also suggests that the unmethylated control group presents more stable hydrogen bonds between interacting pairs.

## Discussion

**Figure 8.**
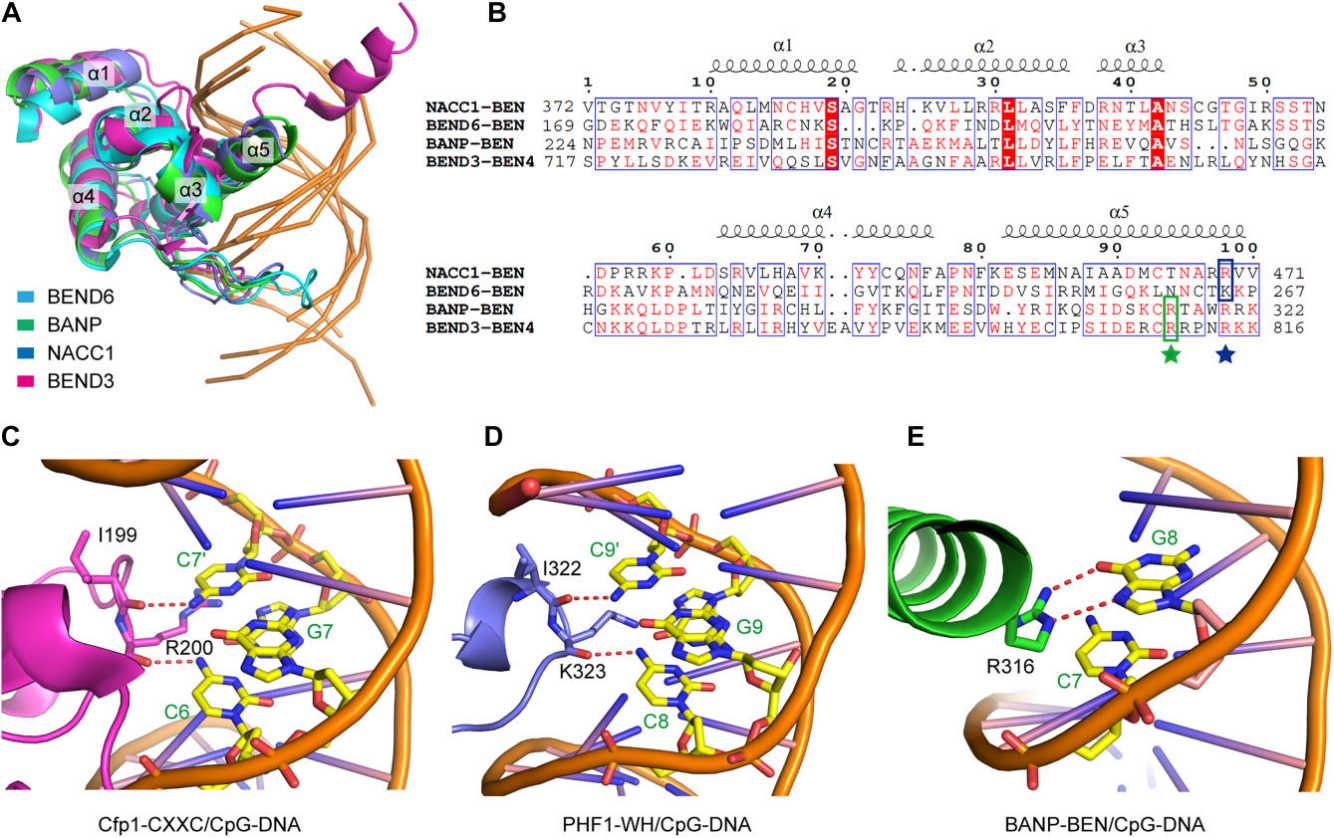
Structural analysis of the DNA recognition mechanism of various CpG motif-binding proteins. (**A**) Structural alignment of solved DNA-bound human BEN domain proteins of BEND3 (PDB entry: 7W27), BEND6 (PDB entry: 7YUN), NACC1 (this study) and BANP (this study). Five α-helices of the BEN domains are labelled. (**B**) Sequence alignments of the BEN domains of the four human BEN domain proteins described in panel (A). The secondary structural elements of the NACC1 BEN domain are shown above the sequences. Key residues that are involved in base recognition for BANP and BEND3 and for NACC1 and BEND6 are highlighted with a box and indicated with a star below, respectively. (**C**) The CXXC domain of Cfp1 (PDB entry: 3QMH) recognizes the cytosines of the CpG motif through two main chain-mediated hydrogen bonds. (**D**) The WH domain of PHF1 (PDB entry: 5XFP) also recognizes the cytosines of the CpG motif through two main chain-mediated hydrogen bonds. (**E**) The BEN domain of BANP (this study) recognizes one cytosine of the CpG motif through stacking with the side chain of an arginine.

The BEN domain is a new type of DBD that has been identified in recent years. Based on the presence or absence of the CpG motifs within the preferred binding sequences, the BEN domain proteins can be divided into two groups. In this study, we solved the crystal structures of DNA-bound BEN domains from both BANP and NACC1, which are representative members of each group of BEN domain proteins. By comparing the structures of the solved human BEN domains from NACC1, BANP, BEND3 and BEND6, we found that the BEN domains bind the target DNA in a very conserved manner (Figure 8A), unlike the WH domains, which bind DNA in a variety of manners (43). A typical BEN domain contains five conserved α-helices. Among all of the available DNA-binding BEN domain structures in humans, the C-terminal helix α5 is mainly responsible for DNA recognition, as it is the only helix that is inserted into the major groove for base-specific recognition (Figure 8A). Helix α2 and the loop between α3 and α4, with the help of the C-terminal helix α5, can strengthen binding through contacts with the phosphate backbone. For the BEN domains from BEND6 and NACC1, as the loop between α3 and α4 is of sufficient length, bases in the minor grooves are also recognized (Figure 8A). Based on the structure and sequence alignments, we found that for CpG-binding proteins, the site in α5 corresponding to the Arg316 residue in BANP is conserved and plays a key role in CpG selection, as has also been shown in BEND3 (Figure 8B). For non-CpG-binding proteins such as NACC1 and BEND6, the site in α5 corresponding to Arg469 in NACC1 seems to be more important in selecting key motifs, as is also shown in BEND6 (Figure 8B). The identification of these two sites may help us to analyse other BEN domain proteins.

CpG islands are hypomethylated and promoter-enriched DNA elements that are found in most mammalian genomes and that regulate gene expression by recruiting a variety of transcription factors. CpG island-binding proteins usually specifically recognize one or several unmethylated CpG motifs while being repelled by CpG motif methylation. The first identified CpG island-binding protein contains a CXXC domain (44), which contains two zinc fingers and recognizes the unmethylated cytosines in the CpG motif through two main chain-mediated hydrogen bonds (44,45) (Figure 7C). As the main chain atoms are positioned very close to the base of the unmethylated cytosine, methylation of this cytosine would cause steric hindrance to disrupt the interaction (Figure 8C). Our laboratory subsequently reported that the WH domain of human PCL proteins is a new type of unmethylated CpG motif-binding domain (46). The WH domain is folded by histone H5 and has been shown to bind a variety of DNA molecules and even RNAs (43). The human PCL proteins, SAMD1 and KAT6A/B all contain a WH domain that recognizes unmethylated CpG motifs (46–48). Although structurally distinct from the zinc finger-containing CXXC domain, the WH domain actually recognizes the unmethylated cytosine through a mechanism that is quite similar to that of the CXXC domain (Figure 8D). Specifically, both unmethylated cytosines in the CpG motif are also recognized by the WH domain through two main chain-mediated hydrogen bonds. The close contact between cytosine and the protein backbone cannot accommodate an added methyl group.

The BEN domain can be grouped into the third class of CpG island-binding domains. As shown for BANP and BEND3, BEN domains prefer to bind unmethylated CpG motifs, which is consistent with their recruitment to CpG islands *in vivo*. Notably, these BEN domains recognize unmethylated cytosines through a mechanism that is quite different from that shown by the CXXC and WH domains. In the structure of the DNA-bound BEN domain of BANP, the unmethylated cytosine of the CpG motif does not form any hydrogen bonds with a residue but is stabilized through stacking with the side chain of an arginine (Figure 8E). Methylation of this cytosine would introduce steric clash with the arginine side chain or the adjacent base, thus establishing the structural basis for methylation sensitivity. Unexpectedly, the sensitivity to methylation is not significant when the BEN domain is in a monomeric state, which may be due to the flexibility of the side chain of arginine or the adjacent DNA bases. Via oligomerization, two or more BEN domains bind the tandem CpG motifs in a coordinated manner, thus achieving a preference for unmethylated DNA substrates. Molecular dynamics simulations further verified that oligomerized BANP tends to form a more stable complex with the unmethylated DNA substrate than it does with its methylated counterpart. Oligomerization through hetero- or self-oligomerization domains frequently occurs in many transcription factors; however, how these oligomerized components function in a coordinated manner remains to be a difficult problem to solve. Our study highlights the importance of oligomerization in regulating the function of DNA-binding proteins and reveals a new mode of DNA recognition in multicomponent complexes. BEN domain proteins are closely related to multiple human diseases. Our study demonstrated that several cancer-related mutations in the BEN domain of NACC1 and BANP disrupt DNA recognition, which may provide some clues for their relevance to human malignancy and may inspire us to identify new therapeutic strategies.

## Supplementary Material

gkae762_Supplemental_File

## Data Availability

The coordinates of NACC1 and BANP with their bound DNA substrates were deposited in the Protein Data Bank (http://www.rcsb.org) with accession numbers 8YZS and 8YZT, respectively.
