# Supplementary material for: Structural basis of DNA recognition by BEN domain proteins reveals a role for oligomerization in unmethylated DNA selection by BANP
Source: Nucleic Acids Res. 2024 Sep 3;52(18):11349–61. doi: 10.1093/nar/gkae762 (PMC11472053; doi:10.1093/nar/gkae762)
Supplement: gkae762_Supplemental_File [file gkae762_supplemental_file.pdf]

Supplementary Data for

Structural basis of DNA recognition by BEN domain proteins reveals a role for oligomerization in unmethylated DNA selection by BANP

Jiahao Ren[1#], Junmeng Wang[1#], Yanpeng Ren[1#], Yuyang Zhang[2,3#], Pengshuai Wei[1], Meng Wang[1], Yimeng Zhang[1], Meng Li[1], Chuyan Yuan[1], Haipeng Gong[2,3*], Junyi Jiang[1*] and Zhanxin Wang[1*]

[1]Key Laboratory of Cell Proliferation and Regulation Biology of Ministry of Education, College of Life Sciences, Beijing Normal University, 19 Xinjiekouwai Avenue, Beijing 100875, China

[2]MOE Key Laboratory of Bioinformatics, School of Life Sciences, Tsinghua University, Beijing, 100084, China

[3]Beijing Frontier Research Center for Biological Structure, Tsinghua University, Beijing, 100084, China

[#]These authors contributed equally to this paper.

[*]Corresponding author: hgong@tsinghua.edu.cn; jiangjy@bnu.edu.cn; wangz@bnu.edu.cn

This PDF file includes the following:

Supplementary Figures S1 to S3
Supplementary Tables S1 and S2

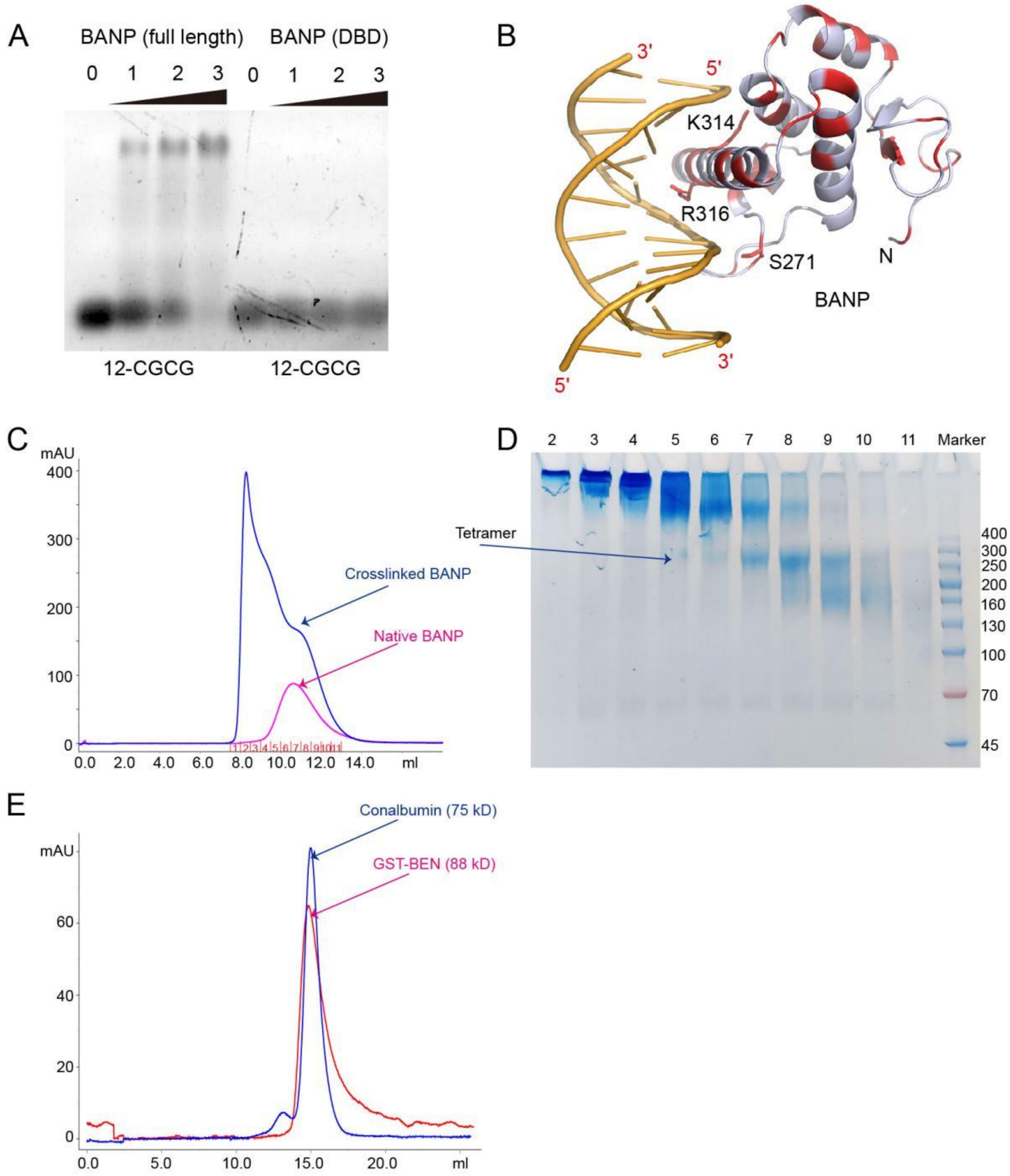

Supplementary Figure S1. Structural and biochemical analysis of BANP. (**A**) EMSA analysis of the full-length and DBD domains of BANP with 12-CGCG DNA. (**B**) Cancer-related mutations mapped onto the BEN domain of BANP. Positions of missense mutations (coloured in red) recorded in the COSMIC database are mapped on the BEN domain of BANP. DNA-binding residues that have mutations are shown in stick models. (**C**) Gel-filtration profiles of the native (pink) and crosslinked full-length BANP. (**D**) SDS–PAGE of the eluted fractions of cross-linked BANP from (C). (**E**) Gel-filtration profiles of GST-BEN and the standard protein conalbumin.

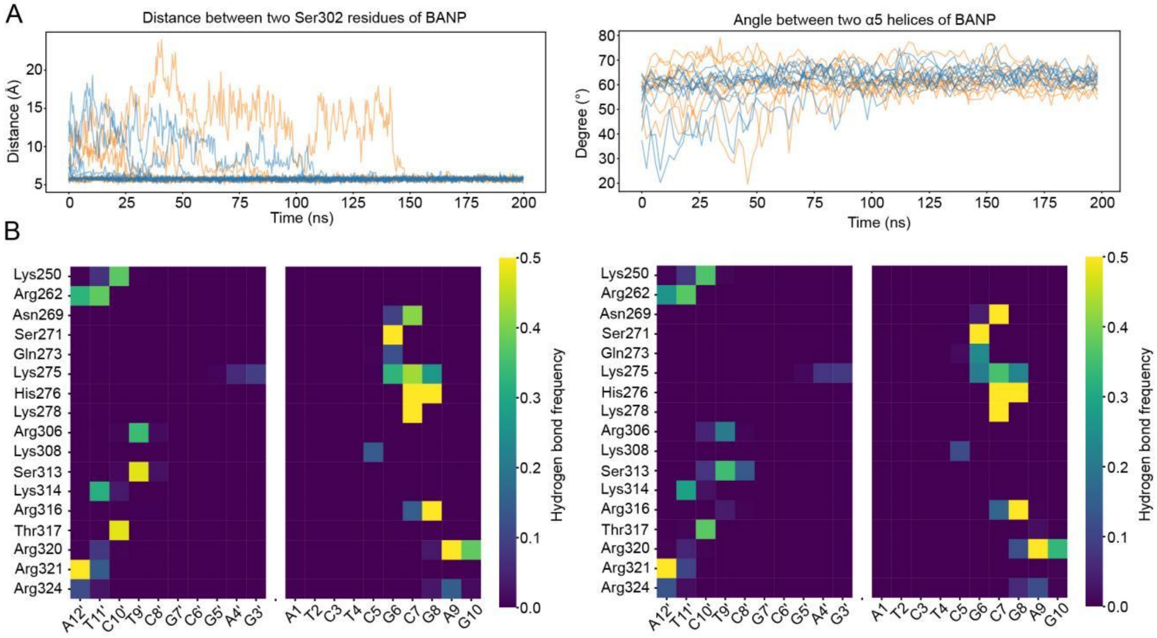

Supplementary Figure S2. Molecular dynamics analysis of the interaction between BANP and CGCG-containing DNA substrates with/without methylation. (**A**) Curves of distances between two Ser302 residues (left) and angles between two α5 helices (right) from two BEN domains of BANP during simulation. As a reference, in the crystal structure, the distance between two Ser302 residues and the angle between two α5 helices are 9.73 Å and 58.6°, respectively. The control and MT groups are coloured blue and orange, respectively. (**B**) Plots of the frequencies of hydrogen bond formation between key residues of BANP and the bases from the DNA substrate in the control (left) and MT (right) groups.

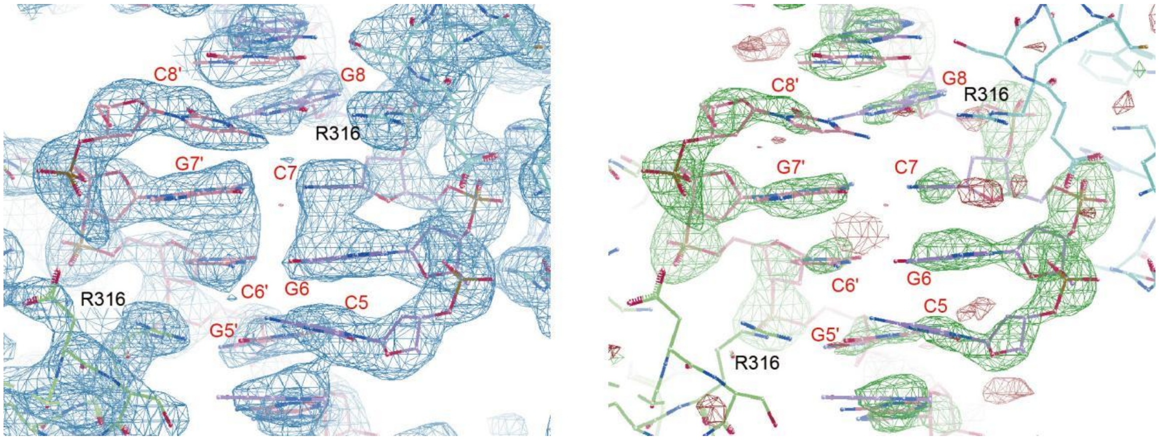

Supplementary Figure S3. Omit electron density maps of the CGCG motif. Left, 2Fo-Fc omit map of the CGCG motif contoured at the 1.0σ level. Right, Fo-Fc omit map of the CGCG motif contoured at the 3.0σ level. Both maps were calculated without the DNA molecules in the initial models. The DNA models from our refined structures are shown in the maps as the references.

Supplementary Table S1. Data collection and refinement statistics

	SeMET-labelled NACC1/DNA complex	BANP/DNA complex
PDB code	8YZS	8YZT
Data collection and refinement		
Beamline	BL18U1	BL18U1
Wavelength (Å)	0.97853	0.97915
Space group	$P2_12_12_1$	$P2_12_12_1$
Cell dimensions		
a, *b*, *c* (Å)	77.13, 83.88, 133.85	58.00, 58.06, 214.38
α, β, γ (°)	90.0, 90.0, 90.0	90.0, 90.0, 90.0
Resolution (Å)	28.83-2.30 (2.36-2.30)*	39.36-2.58 (2.70-2.58)
R_{merge}	0.113 (1.096)	0.139 (0.591)
I/σ	21.4 (1.8)	16.6 (2.0)
Completeness (%)	100.0 (100)	99.1 (94.1)
Redundancy	13.1 (13.5)	8.0 (5.3)
No. reflections	38861	23248
R_{work}/R_{free}	0.213/0.248	0.225/0.305
No. atoms		
Protein	3834	3921
DNA	888	524
water	132	15
B-factors (Å^2)		
Protein	53.3	58.3
DNA	41.1	55.8
water	44.8	50.5
R.m.s. deviations		
Bond lengths (Å)	0.009	0.009
Bond angles (°)	1.093	0.984
Ramachandran Plot		
Favoured residues	450	453
Allowed residues	21	18
Outliers	0	0

*Values in parentheses are for highest-resolution shell

Supplementary Table S2. ITC-based binding parameters for complex formation between different DNA substrates and wild-type or mutant of NACC1 or BANP proteins.

DNA	Protein Sample	N value	K_D (μM)	ΔH (kcal/mol)
12-CATG	NACC1-BEN WT	0.91	1.3 ± 0.2	5.26 ± 0.08
12-CATG	NACC1-BEN R462A	0.84	4.6 ± 0.4	5.86 ± 0.10
12-CATG	NACC1-BEN N466A	0.84	2.9 ± 0.4	5.79 ± 0.11
12-CATG	NACC1-BEN R421A	0.85	4.6 ± 0.6	5.61 ± 0.12
12-CATG	NACC1-BEN R472A	0.86	8.6 ± 1.4	5.91 ± 0.23
12-CATG	NACC1-BEN R429A	0.89	34.7± 4.5	5.24 ± 0.38
12-CATG	NACC1-BEN R468A	N.A.	N.A.	N.A.
12-CATG	NACC1-BEN R469A	N.A.	N.A.	N.A.
12-CGCG	BANP-BEN WT	0.52	2.6 ± 0.4	-31.73 ± 0.73
12-mCGCG	BANP-BEN WT	0.49	5.6 ± 0.7	-24.59 ± 0.82
12-mCGmCG	BANP-BEN WT	0.49	5.7 ± 0.9	-24.36 ± 0.95
12-CGCG	BANP-BEN K314A	0.47	25.2 ± 3.8	-6.08 ± 0.57
12-CGCG	BANP-BEN K278A	0.40	35.6 ± 4.1	-16.50 ± 2.66
12-CGCG	BANP-BEN S313A	0.63	7.9 ± 1.1	-12.91 ± 0.44
12-CGCG	BANP-BEN S310A	0.46	12.8 ± 0.9	-19.53 ± 0.54
12-CGCG	BANP-BEN R316A	0.62	16.8 ± 1.6	-21.10 ± 1.10
12-CGCG	BANP WT full-length	0.25	3.4 ± 0.6	-11.0 ± 0.56
12-mCGCG	BANP WT full-length	0.25	4.9 ± 1.4	-2.78 ± 0.30
12-mCGmCG	BANP WT full-length	N.A.	N.A.	N.A.
12-CGCG	BANP CC-BEN (1-360)	0.30	2.9 ± 0.7	-14.35 ± 0.72
12-mCGCG	BANP CC-BEN (1-360)	0.26	4.3 ± 0.8	-3.55 ± 0.52
12-mCGmCG	BANP CC-BEN (1-360)	N.A.	N.A.	N.A.
12-CGCG	BANP ΔCC (90-519)	0.31	3.4 ± 0.5	-23.3 ± 0.79
12-mCGCG	BANP ΔCC (90-519)	0.51	5.6 ± 0.9	-16.58 ± 0.81
12-mCGmCG	BANP ΔCC (90-519)	N.A.	N.A.	N.A.
12-CGCG	BANP BEN-DBD (205-519)	0.31	3.1 ± 0.3	-28.54 ± 0.95
12-mCGCG	BANP BEN-DBD (205-519)	0.50	8.3 ± 0.6	-22.06 ± 0.42
12-mCGmCG	BANP BEN-DBD (205-519)	N.A.	N.A.	N.A.
12-CGCG	BANP GST-BEN	0.23	2.3 ± 0.4	-16.36 ± 0.70
12-mCGCG	BANP GST-BEN	0.24	3.5 ± 0.6	-5.99 ± 0.41
12-mCGmCG	BANP GST-BEN	N.A.	N.A.	N.A.
